# The hospital burden of intergenerational contact with the welfare system: A whole-of-population liked data study.

**DOI:** 10.23889/ijpds.v7i3.1856

**Published:** 2022-08-25

**Authors:** Alexandra Procter, Catherine Chittleborough, Rhiannon Pilkington, Odette Pearson, Alicia Montgomerie, John Lynch

**Affiliations:** 1 The University of Adelaide; 2 South Australian Health and Medical Research Institute

## Objectives

Intergenerational welfare contact (IWC) is a policy issue due to personal and social costs of ‘entrenched disadvantage’ across generations, yet few studies have quantified the burden of IWC on health. We examine the proportion of children who experienced IWC and other welfare contact (WC) types; and estimate cause-specific hospital burden.

## Approach

A whole-of-population linked administrative data study of children in South Australia born 1991-1995 (n=94,358), followed from birth up to age 20 years and their parent/s (n=143,814). The study used de-identified data from the Better Evidence Better Outcomes Linked Data platform including data from Australian Government Centrelink (welfare payments), birth registration, perinatal birth records, and inpatient hospitalisations. Children were classified as: No WC, parent only WC, child only WC, or IWC. Cause-specific hospitalisation rates and cumulative incidence were estimated by age and WC group.

## Results

Using Australian Government Centrelink data, WC was defined as parent/s receiving a means-tested welfare payment (low-income, unemployment, disability or caring) when children were aged 11-15, or children receiving payment at ages 16-20. IWC was WC occurring in both parent and child generations. IWC affected 34.9% of children, who had the highest hospitalisation rate (133.5 per 1,000 person-years) compared to no WC (46.1 per 1,000 person-years), parent WC (75.0 per 1,000 person-years), and child only WC (87.6 per 1,000 person-years). Of all IWC children, 43.0% experienced at least one hospitalisation between 11-20 years, frequently related to injury, mental health, and pregnancy.

## Conclusion and Relevance

Children experiencing IWC represent a third of the population aged 11-20. Compared to children with parent-only WC, IWC children had 78% higher hospitalisation rates from age 11 to 20, accounting for over half of all hospitalisations in this age group. Frequent IWC hospitalisation causes were injuries, mental health, and pregnancy.

